# Design and manufacture of 3D cell culture plate for mass production of cell-spheroids

**DOI:** 10.1038/s41598-019-50186-0

**Published:** 2019-09-27

**Authors:** Dongkyoung Lee, Shiva Pathak, Jee-Heon Jeong

**Affiliations:** 1 Department of Mechanical and Automotive Engineering, Kongju National University, Cheonan, 31080 South Korea; 2 College of Pharmacy, Yeungnam University, Gyeongsan, Gyeongbuk 38541 South Korea

**Keywords:** Biophysical methods, Biomaterials

## Abstract

A 3D cell culture is preferred to 2D cell culture since it allows cells to grow in all directions *in vitro*, similar to how they would *in vivo*. 3D cell culture plates currently used in tissue engineering research have limited access to control the geometry. Furthermore, 3D cell culture plate manufacturing methods are relatively complex, time-consuming, labor-intensive, and expensive. Therefore, a design and manufacturing method, which has relatively low cost, high throughput, and high size flexibility, is proposed. Cell culture plate was fabricated by computer aided design and manufacturing software using polydimethylsiloxane as a plate constituent. With the successfully-developed 3D cell culture plate, the morphology and viability of the cultured mesenchymal stem cells were tested.The mesenchymal stem cells seeded on the newly-fabricated 3D cell culture plate aggregated to form 3D spheroids within 24 h of incubation and well-maintained their viability. Thus, due to the capacity of mass production of the cell spheroids with a desired cell viability, the newly-fabricated plate has a great promise to prepare 3D cell spheroids for experimental as well as clinical applications.

## Introduction

Scientists in the field of regenerative medicine and tissue engineering are thriving to construct tissue and organ substitutes that can restore normal function in diseased or injured tissues^1–5^. Attempts have been implemented by applying the principles of nanotechnology, material science, and bioengineering to control biological, biophysical and/or biomechanical parameters of the constructs^6–8^. In regenerative medicine and tissue engineering, 3D cell culture is preferred to 2D/monolayer culture because it allows cells to grow in all directions *in vitro*, which is similar to how they would *in vivo*^9–11^. Hanging drop method is used as the most common way of 3D culture in tissue engineering^12,13^. This method employs the application of gravity-assisted spontaneous aggregation of cells on a drop of media that hangs on the upper lid of a cell culture dish. Due to the involvement of labor intensiveness and difficulty in scale-up, several alternative ways to prepare 3D spheroids have been investigated.

Many tissue engineering studies have been carried out with various cell culture plates^14,15^. However, these studies have limited access to control the geometry of the cell culture plates. Thus, flexibility and controllability of the 3D cell culture plates in terms of size are poor. Furthermore, above-mentioned techniques of fabricating concave 3D cell culture plates are relatively complex, time-consuming, labor-intensive, and expensive. Therefore, cost-effectiveness, high throughput, and size flexibility of 3D cell culture plates are required to accelerate the tissue engineering research. This study aimed to develop a 3D culture plate with the easily adjustable manufacturing design process for cell culture-friendly environment and mass-production. Therefore, 10 × 10 embossing type cell culture plates with the diameter of 2 mm for each well was designed and manufactured. Moreover, viability of the mesenchymal stem cells cultured in the developed plate was tested.

## Results

### The production process

**Figure 1:**
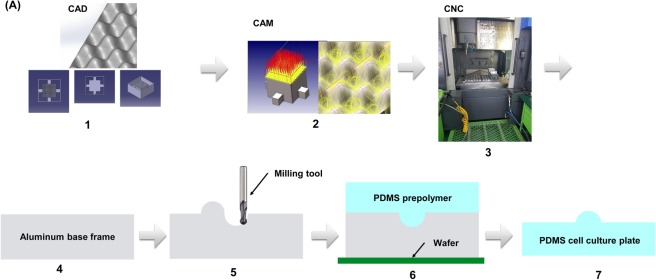
The production process of the cell culture plate. (**A**) The design of base frame was conducted using CAD software (CATIA®). CNC tool path and programming code was created from CAM software (hyperMill®). Finally, CNC programming was transferred into CNC machining center (SIRUS-UM) and the base frame was machined. PDMS was poured and filled into the machined base frame. After PDMS was dried and fully hardened, it was detached from the base frame.

Figure 1 shows the overall process for the production of the 3D cell culture plate. The base frame was designed by computer-aided design software. Manufacture of the culture plate was performed by computer-aided manufacturing software. To prepare the culture by the above design, polydimethylsiloxane (PDMS) was filled into the machined base frame and dried. After the sufficient hardening of the PDMS, the cell culture PDMS plate was mechanically detached from the base frame.

### Base frame design and manufacture

A base frame was used as a mold where the PDMS was poured and dried. The material of the base frame has a wide choice. However, there should be no chemical interaction between PDMS and the base frame. Hence, aluminum alloy was chosen due to its wide use, low density, corrosion resistance property, and no chemical reaction to PDMS. To make the cell culture plate for mass production, a concave shape was proposed since the sharp edge such as a vertex of a triangular pyramid is hardly manufactured by the machining process. Furthermore, the concave geometry was similar to the shape formed by the hanging drop method.

**Figure 2:**
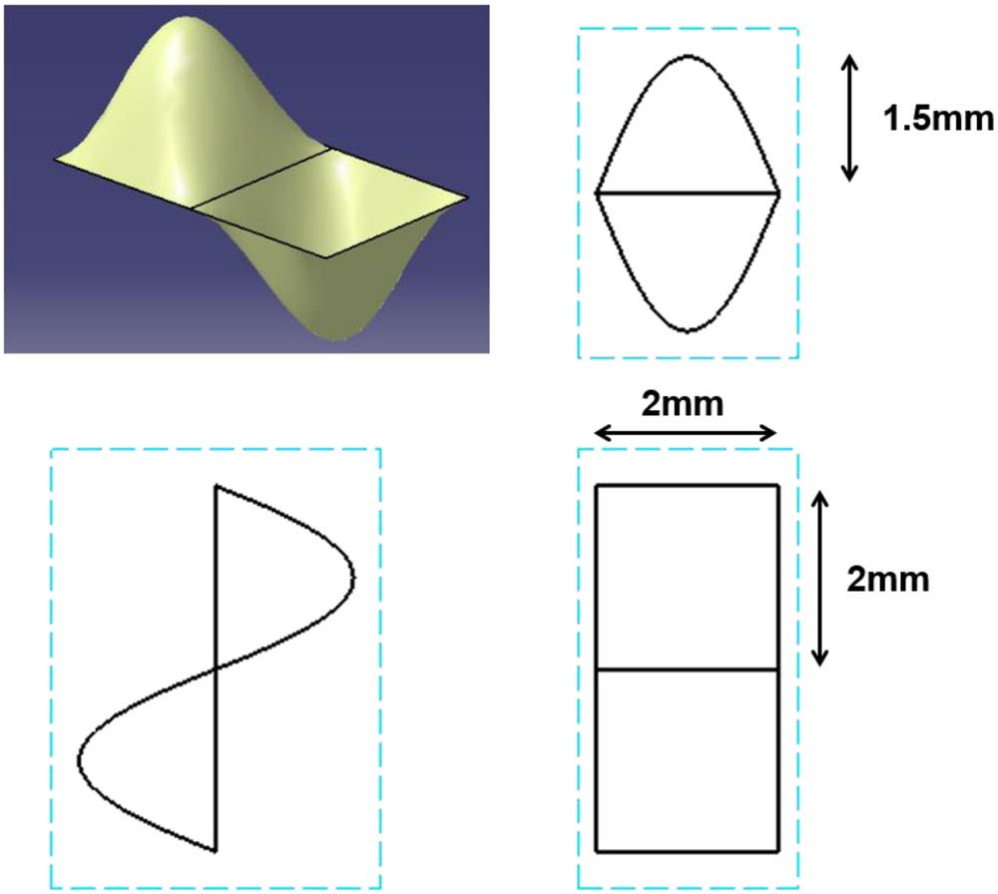
The geometry of the single well and one module. The positive single well is formed by using ‘fill surface function’, where the center of the square is pulled into the upper direction and pyramid type single well is created. One positive and negative well forms one module.

**Figure 3:**
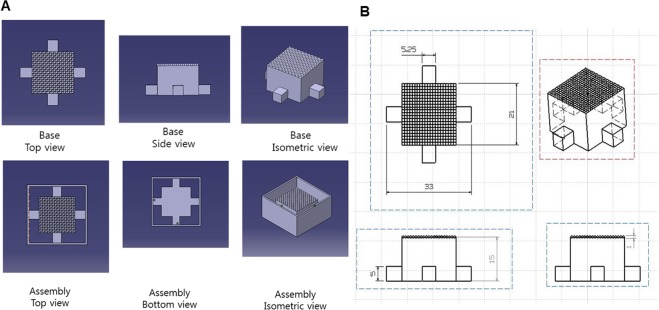
Isometric, side, and top view of the base frame with scales. (**A**) A base frame geometry from CAD. Each edge of the positive single well was enclosed by the negative single well and the positive single well was placed in diagonally opposite place. This pattern was repeated 10 times in the horizontal and vertical direction (**B**) Size and scale are described in 2D drawings.

A CAD software was used to design the base frame. The geometry of a single well and one module is shown in Fig. 2. The bottom geometry was square and the length was 2 × 2 mm. The height was 1.5 mm. The positive single well was formed by using ‘fill surface function’ in the software (CATIA). The center of the square was pulled into the upper direction and pyramid type single well was created. Each edge of the positive single well was enclosed by the negative single well and the positive single well was placed in diagonally-opposite place. This pattern was repeated 10 times in the horizontal and vertical direction. Isometric, side, and top view of the base frame are shown in Fig. 3.

The auxiliary parts were designed to help separate the base frame and the cell culture plate easily. The auxiliary parts were composed of two parts as shown in Fig. 3. One was the inner frame and the other was the outer frame. The inner frame contained the repeated single wells on the top and a cube shape was formed under the repeated single wells. The outer frame was used to hold a liquid PDMS and make a wall.

**Figure 4:**
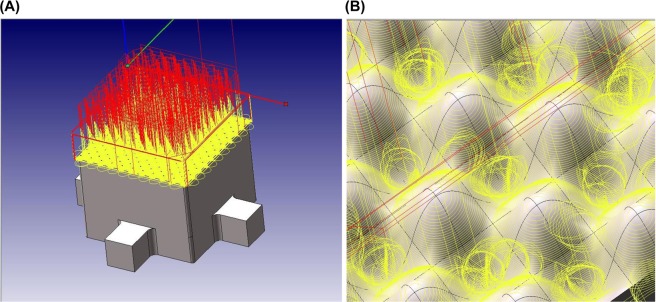
CNC machining path created by CAM software. Tool paths with yellow and red colors indicate machining path and moving path, respectively. CAM converts this visual tool path into CNC programming code running a CNC machining center.

After this design, the base frame was manufactured. Although there are many manufacturing methods such as laser-aided manufacturing^16–24^, electron beam cutting^25–27^, a CNC machining center was selected due to its availability of well-developed hardware and software. The tool parameters used for the CNC machining is shown in Table 1. The ball end mills with a diameter of 0.6 mm and 1.0 mm were used. The ball end mill with a diameter of 0.6 mm rotated 15,000 rev/min and the feed rate was 800 mm/min. The ball end mill with a diameter of 1.0 mm rotated 15,000 rev/min and the feed rate was 1000 mm/min. The CNC machining path provided by CAM software is shown in Fig. 4. Yellow and red colors indicate the machining and moving tool path, respectively. These paths were then transformed to the CNC programming code, or the G-code, which is CNC programming language to operate the CNC machine.

**Table 1:** CNC machining tool parameters.

Ball end mill 0.6 ∅		Ball end mill 1.0 ∅	
**RPM**	**Feed**	**RPM**	**Feed**
15,000 (rev/min)	800 (mm/min)	15,000 (rev/min)	1,000 (mm/min)

### Base frame and 3D cell culture plate

**Figure 5:**
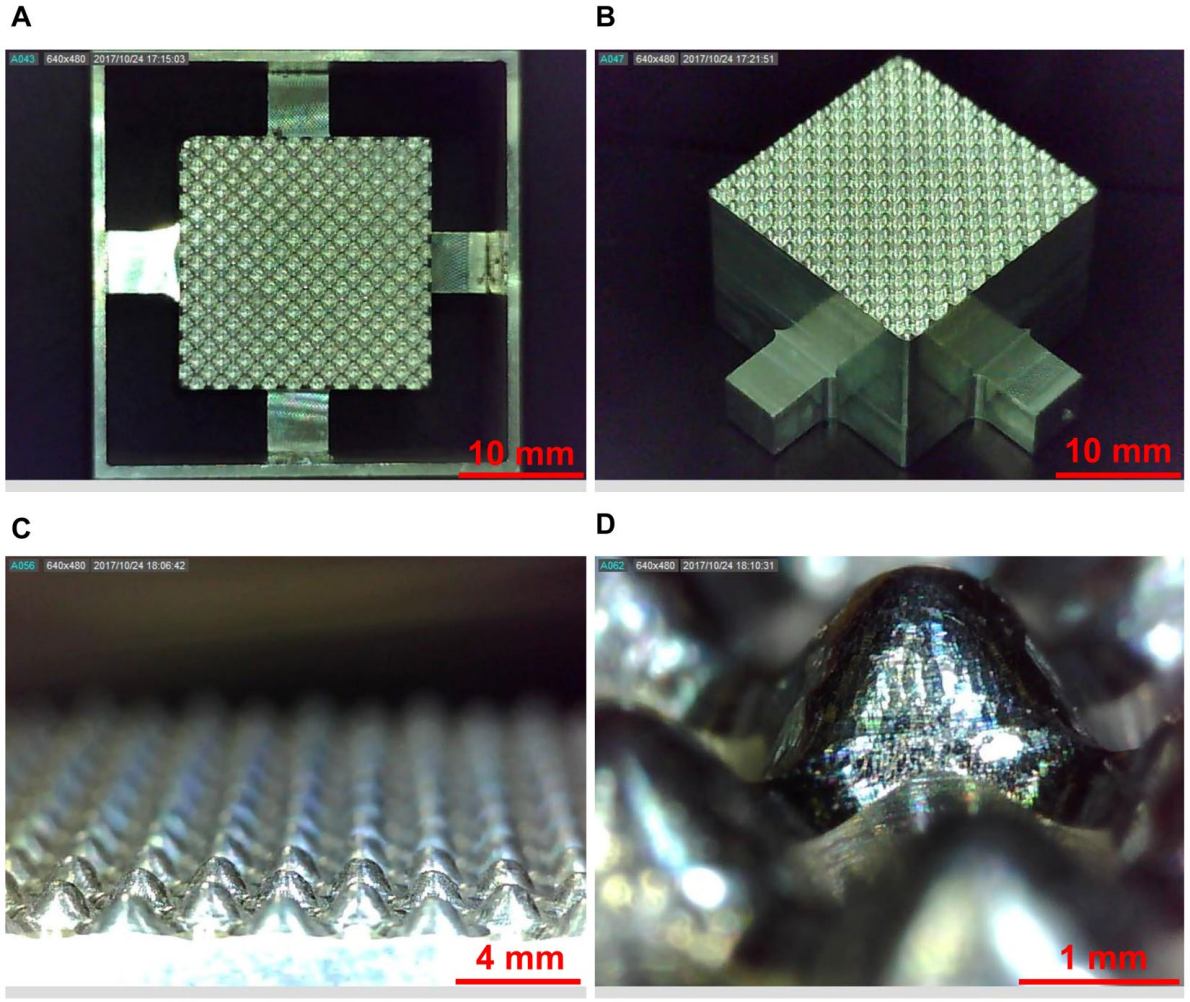
The final product of the base frame and a single well. (**A**) The assembly of the base frame and auxiliary showed a perfect match as designed from the CAD software. (**B**) Well-manufactured pattern showed a clean edge with no defects. (**C**) The magnified base frame showed a repetition of the positive single well and its repetition was well-aligned. (**D**) The smooth surface of the positive single well was shown and the size of the single well showed a match with the design.

**Figure 6:**
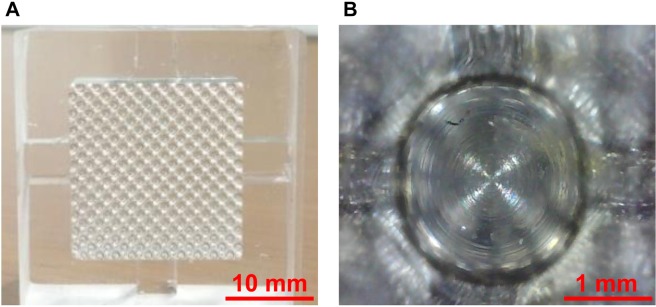
The final product of the 3D cell culture plate and a single well. (**A**) Embossing type 3D cell culture plate was fabricated based on the base frame. The embossing morphology was well-transferred into the PDMS. (**B**) The single well was also transferred into PDMS, and all single wells maintained a desired quality.

The final product of the base frame is shown in Fig. 5. The base frame showed a perfect match as designed from the CAD software. The size of the single well was measured and it also matched with the design. The final product of the 3D cell culture plate is shown in Fig. 6. The pattern was successfully transferred. The 3D cell culture plate showed a good agreement with the design. The size of the single well was measured and it also showed a good agreement. Therefore, it was confirmed that the suggested design and manufacturing process can successfully provide the base frame. Each plate contained 100 microwells and is capable of forming 100 spheroids at a single time.

### Cell morphology and viability

**Figure 7:**
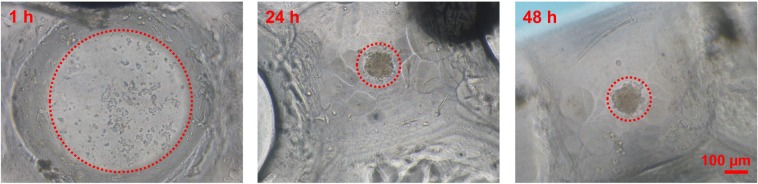
Morphology of MSC on the newly-fabricated 3D culture plate at different time points. At 1 h, the MSC showed a monolayer fashion. At 24 h, the MSC aggregated forming 3D structure. No change in the morphology of spheroids was observed during the observation period. Magnification: 100, Scale bar: 100 µm.

The morphology of mesenchymal stem cells (MSC) on the newly-fabricated plate is shown in Fig. 7. After 1 h of seeding, the MSC seemed attached on the surface of the plate in a monolayer fashion. When the morphology of the cells was observed after 24 h of incubation, most of the cells aggregated resulting in the formation of spheroids. This indicates the possible involvement of cell migration during incubation to form compact 3D structure. Incubating cells for more than 24 h did not affect the cellular morphology. Thus, we concluded that the spheroids can be effectively formed within 24 h of incubation. This method is advantageous to the conventional hanging drop method, which takes a relatively long time to form spheroids^28^. Thus, the newly-fabricated plate can be used to overcome the shortcomings of hanging drop method such as labor intensiveness, longer time for spheroid formation, difficulty in mass production, and the impossibility of media replacement during the process of spheroid formation.

**Figure 8:**
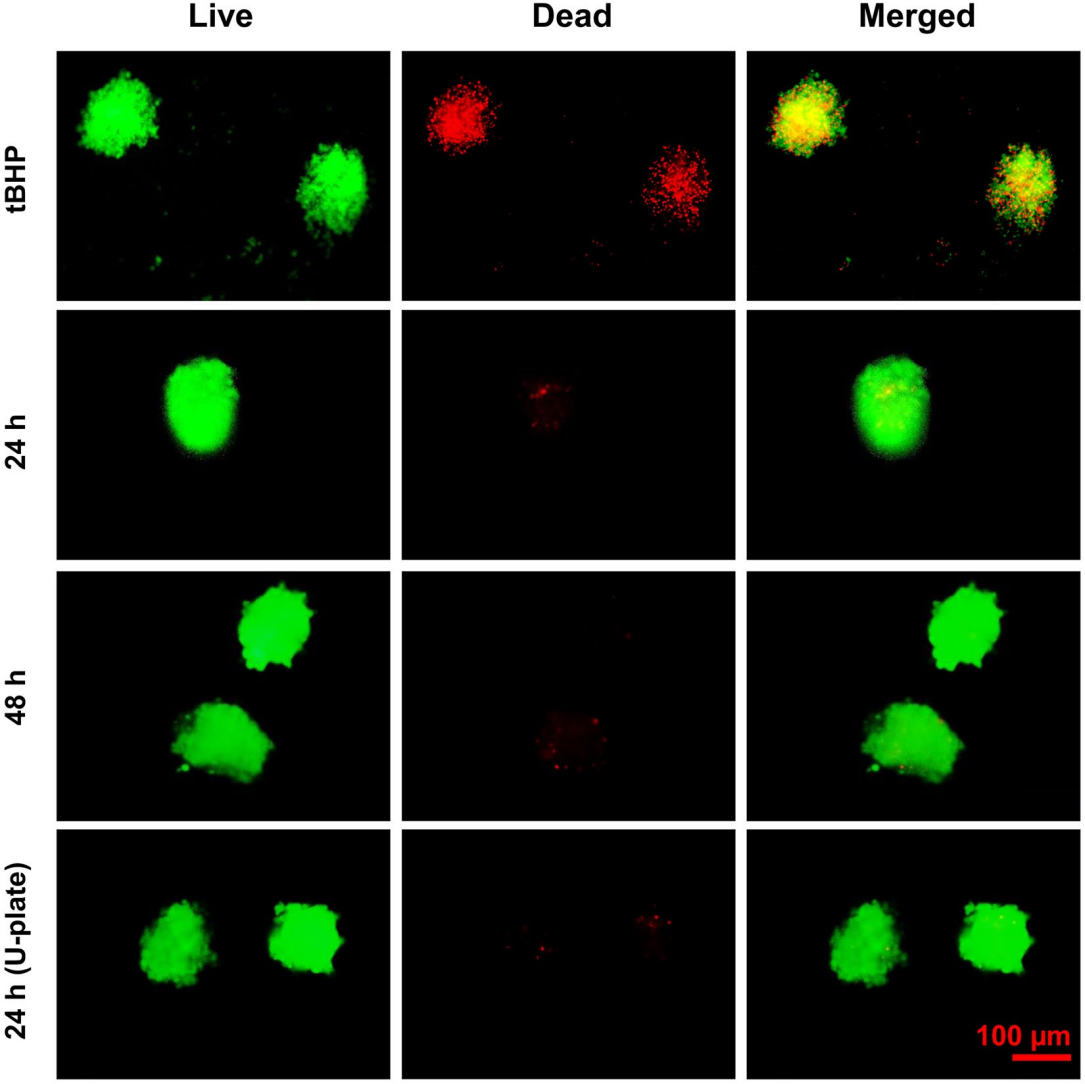
Live/Dead assay for the determination of viability of the MSC spheroids. The spheroids (retrieved after 24 h) were treated with 400 µM tert-Butyl hydroperoxide (tBHP) for 24 h and used as positive control. Live/dead staining was performed at 24 h and 48 h of incubation. Spheroid viability was compared with that of the spheroids prepared by using ultralow attachment U-shaped 96-well plate (Cornings). Green fluorescence indicates AO stain in the live cells and red fluorescence indicates the PI stain in the dead cells. Magnification: 200, Scale bar: 100 µm.

Live/dead staining showed that the viability of cells during spheroid formation was preserved. An intense green fluorescence signal and a very weak red fluorescence signal indicates a well-preserved viability of cells in the spheroids formed by using the newly-fabricated plate at 24 and 48 h of incubation. In contrast, tert-Butyl hydrogenperoxide (tBHP)-treated MSC spheroids showed an intense red fluorescence indicating a massive cell death. Interestingly, the viability of the spheroids formed by using the newly-fabricated plates was similar to that of the spheroids formed by using a commercially-available U-plate for spheroid production (Fig. 8). Thus, due to the capacity of mass production of the cell spheroids with good cell viability, the newly-fabricated plate has a great promise to prepare 3D cell spheroids for experimental as well as clinical applications.

## Discussion

In the current study, we propose a newly-fabricated 3D cell culture plate with the aid of CAD/CAM for the large-scale production of 3D cell spheroids. Our method of forming the microfabricated culture plates led to the generation of microwells with precise dimensions. The newly-fabricated microwell effectively formed 3D clusters of MSC without causing a significant loss of viability. Unlike the hanging drop method which involves labor intensive procedure, microfabrication is an advanced technique which involves spheroid formation assisted by micropatterns of defined geometry. In addition, microfabrication leads to rapid formation of spheroids with well-controlled size in a larger scale compared to the conventional hanging drop method.

Previous studies have reported the use of a various microfabrication techniques for the preparation of cell spheroids. In this regard, Lee *et al*. suggested *in situ* islet spheroid formation and encapsulation using molds. Single cells were seeded onto a PDMS-based micro concave mold to form 3D aggregates. To encapsulate the 2 week-cultured spheroids, the Collagen-Alginate Composite (CAC) solution was applied and spread over the concave mold. Then, a porous membrane was spread to cover the solution-filled concave wells. A calcium chloride solution was pipetted on to the porous membrane and incubated. Finally, the CAC containing islet spheroids was detached. With the use of 300 μm concave wells, spheroids having controlled-size range were produced^29^. In another study, a straightforward strategy of a high throughput concave microwell array was presented by Shi *et al*.^30^. Polydimethylsiloxane (PDMS) concave microwells were fabricated by using negative photoresist SU-8. The SU-8 polymer was initially spin-coated on the surface of a pre-baked glass substrate and then exposed to UV light. This SU-8 coated glass substrate was immersed in ethyl lactate and heated to produce the mold structure with a concave configuration. With the concave SU-8, the first PDMS layer with convex structures was fabricated using soft lithographic technology. The first PDMS layer was treated by PF-127 solution to modify the surface and then heated to remove the water. And then, new PDMS was poured over the first PDMS layer. After full polymerization, the PDMS layer was peeled off to form concave microwell. Using this concave microwell, 3D spheroids, aimed to facilitate the cartilage-specific phenotype and function maintenance, were constructed^30^.

Scaffold surface-features are important regulators of stem cell performance and endurance in tissue engineering applications. Graziano *et al*.^31^ studied the effect of concave pit-containing scaffold surfaces on stem cell-derived osteoblast performance. To obtain scaffold with two surfaces texturing, poly(lactic-*co*-glycolic acid) (PLGA) was carved to form membranes. Firstly, a smooth surface was obtained using PLGA membrane. And then, a concave texturing was obtained after treating the membrane with *N*-Methyl-2-pyrrolidone (NMP), which controls subtractive pits. In the current study, the PDMS-based microwell formed loosely-attached MSC spheroids probably because of the hydrophobic nature of PDMS. Thus, the spheroids formed in the PDMS microwell were easily retrievable after predefined hours of incubation without any significant disruption of cell morphology during the harvesting process. Miko *et al*. experimentally verified the cusp height and surface roughness (Ra), using 3D milling rounded surfaces, and reported a ‘Ra’ value ranging 0.53–3.79 µm^32^. When we compared the dimensions of one microwell, the roughness would be 0.0265–0.1895. Therefore, the current manufacturing technique produces 3D spheroid-producing culture plates with a reasonable roughness.

Spheroid-based 3D liver-on-a-chip and brain-on-a-chip^33,34^ were developed to investigate hepatocyte-hepatic stellate cell interactions and to apply in an *in vitro* model of Alzheimer’s disease, respectively. To construct the concave plate, a 50 cylindrical well array in a backbone PDMS chamber was prepared using a standard soft-lithography process. Liquid PDMS was then poured and polymerized by thermal curing on the backbone PDMS having a 50 cylindrical well array. Due to the surface tension, the liquid PDMS formed a concave microwell. Most of the culture plates reported previously for tissue engineering purposes have limited access to control the geometry of the cell culture plates and have less control over the size of the plates. Moreover, the existing techniques are relatively complex, time-consuming, labor-intensive, and expensive. In the present study, we report a simple and cost-effective method for the fabrication of 3D culture plates with a high-size flexibility. In addition, the current method offers an advantage of manufacturing the culture plates of various dimensions with desired width and depth of each well. This method can be applied for rapid forming of 3D spheroids of various cell-types for laboratory and clinical investigations. In addition, this method can be applied for forming 3D spheroids of other cell types such as hepatocytes and various tumor cells.

## Conclusion

To provide design flexibility and cost-effective manufacturing process, this study suggests the design and manufacturing method with PDMS to make 3D cell culture plate for mass production of cell spheroids. Based on the suggested design and manufacturing process, the 3D cell culture plate can be fabricated easily and adaptively with high geometric flexibility. The CAD software and CAM/CNC machining can be successfully used for designing and manufacturing the base frame for cell culture plate, respectively. In addition, the preserved cell viability after culturing on the suggested 3D cell culture plate demonstrated the applicability of the newly designed plate for tissue engineering approaches.

## Materials and Methods

### Design and manufacture of culture plate

The design and manufacturing process uses a molding process. The production of the cell culture plate for mass production is divided into two parts: (1) Base frame design and manufacturing process, (2) Cell culture plate fabrication process. First, a base frame was designed by Computer Aided Design (CAD) software (CATIA®, Dassault Systems, Velizy-Villacoubly, France) and then manufactured by Computer Aided Manufacturing (CAM) software (hyperMill®, OPEN MIND Technologies AG, Wessling, Germany) and Computer Numerical Control (CNC) machining center (SIRUS-UM, Hwacheon, Seoul, Korea).

### Cell culture plate fabrication process

Polydimethylsiloxane (PDMS) was chosen because it is optically clear, inert, non-toxic, and non-inflammable. With this material, the cell culture plate fabrication process is described as follows. First, a silicon wafer was placed at the bottom to hold the PDMS. Second, the base frame was placed on the top of the silicon wafer. And then, the liquid PDMS was filled into the base frame and dried. In this process, the silicone elastomer and curing agent were mixed in a weight ratio of 10:1 and stirred for approximately 3 minutes so that liquid PDMS mixture was formed. During the stirring, many air bubbles were formed in the liquid PDMS mixture. To remove air bubbles, degassing process was repeated 2~3 times for approximately 1 h in a desiccator by a vacuum pump, and then, the mixture was poured along the wall of the base frame to prevent additional air bubble formation. The liquid PDMS mixture contained in the base frame was dried. A dryer maintaining the uniform temperature distribution, which was driven by convection flow, was used for 30 minutes with the temperature of 120 °C. After the drying process, the 3D cell culture plate was detached from the base frame and unnecessary parts were removed.

### Cell culture and preparation of 3D spheroids

Human adipose-derived mesenchymal stem cells (MSC) were purchased from Stemore (Incheon, Republic of Korea). The MSC at passage 8 were used in the study. The cells were cultured in α-MEM (Hyclone, South Logan, Utah, USA) supplemented with 10% of fetal bovine serum (FBS; Gibco, Grand Island, NY, USA) and 1% of penicillin/streptomycin (GenDEPOT, Barker, TX, USA) at 37 °C in a humidified atmosphere containing 5% of CO_2_. For 3D spheroid formation, 1.21 × 10^5^ cells were suspended in 2.5 mL of the FBS-supplemented media and seeded on the embossing plate. Afterwards, the cell-seeded plates were cultured at 37 °C in a humidified atmosphere. No additional fresh media replacement was required during the culture period.

### Assessment of cell viability

Cell viability was assessed by Live/Dead assay using acridine orange (5 mg/mL) and propidium iodide (3 mg/mL) (Sigma-Aldrich, St. Louis, MO, USA). Briefly, the stem cell spheroids were collected and centrifuged to remove the medium. The spheroids were then suspended in 1 mL of PBS containing 2 μL of acridine orange and 1 μL of propidium iodide. The suspension was then kept at room temperature for 15 min in dark. Afterward, the spheroids were washed two times with PBS to remove the unbound dyes, resuspended in α-MEM, and observed under a fluorescence microscope (Nikon Eclipse Ti, Melville, NY, USA). The live and dead cells were observed under green and red channels, respectively, using exposure time of 200 milliseconds.
